# Video Relay Interpretation and Overcoming Barriers in Health Care for Deaf Users: Scoping Review

**DOI:** 10.2196/32439

**Published:** 2022-06-09

**Authors:** Minerva Rivas Velarde, Caroline Jagoe, Jessica Cuculick

**Affiliations:** 1 Department of Radiology and Medical Informatics Faculty of Medicine University of Geneva Geneve Switzerland; 2 Department of Clinical Speech & Language Studies Trinity College Dublin University of Dublin Dublin Ireland; 3 Department of Liberal Studies National Technical Institute for the Deaf Rochester Institute of Technology Rochester, NY United States

**Keywords:** deafness, disability, accessibility, communication, video, remote interpretation, health care, system, deaf users, sign language, interpreter, medical interpretation, mobile phone

## Abstract

**Background:**

Persons who are deaf are more likely to avoid health care providers than those who can hear, partially because of the lack of means of communication with these providers and the dearth of available interpreters. The use of video remote interpretation, namely the video camera on an electronic device, to connect deaf patients and health providers has rapidly expanded owing to its flexibility and advantageous cost compared with in-person sign language interpretation. Thus, we need to learn more about how this technology could effectively engage with and respond to the priorities of its users.

**Objective:**

We aimed to identify existing evidence regarding the use of video remote interpretation (VRI) in health care settings and to assess whether VRI technology can enable deaf users to overcome barriers to interpretation and improve communication outcomes between them and health care personnel.

**Methods:**

We conducted a search in 7 medical research databases (including MEDLINE, Web of Science, Embase, and Google Scholar) from 2006 including bibliographies and citations of relevant papers. The searches included articles in English, Spanish, and French. The eligibility criteria for study selection included original articles on the use of VRI for deaf or hard of hearing (DHH) sign language users for, or within, health care.

**Results:**

From the original 176 articles identified, 120 were eliminated after reading the article title and abstract, and 41 articles were excluded after they were fully read. In total, 15 articles were included in this study: 4 studies were literature reviews, 4 were surveys, 3 were qualitative studies, and 1 was a mixed methods study that combined qualitative and quantitative data, 1 brief communication, 1 quality improvement report, and 1 secondary analysis. In this scoping review, we identified a knowledge gap regarding the quality of interpretation and training in sign language interpretation for health care. It also shows that this area is underresearched, and evidence is scant. All evidence came from high-income countries, which is particularly problematic given that most DHH persons live in low- and middle-income countries.

**Conclusions:**

Furthering our understanding of the use of VRI technology is pertinent and relevant. The available literature shows that VRI may enable deaf users to overcome interpretation barriers and can potentially improve communication outcomes between them and health personnel within health care services. For VRI to be acceptable, sign language users require a VRI system supported by devices with large screens and a reliable internet connection, as well as qualified interpreters trained on medical interpretation.

## Introduction

### Background

Accessible information and communications technology (ICT), mobile phones, and tools such as video remote interpretation (VRI) aim to enable effective communication between persons who are D/deaf (“Deaf” refers to the linguistic minority while “deaf” refers to persons with hearing impairment) and hard of hearing and those who use sign language as their first language (hereafter, deaf or hard of hearing [DHH] sign language users) and health care personnel. VRI refers to a video camera on an electronic device, either a computer or tablet, that is used to connect patients and health providers with a sign language interpreter via video call. Its use has rapidly expanded owing to its flexibility and advantageous cost compared with in-person sign language interpretation [1]. The cost-efficiency of such technology is a serious concern given that 80% of the DHH population live in low- and middle-income countries (LMICs), where resource constraints tend to limit the availability of qualified sign language interpreters [2]. VRI aims to overcome communication barriers in health care. DHH persons are more likely to avoid health care providers than those who can hear, partially because of the lack of means of communication with these providers and the dearth of available interpreters [3,4]. Even if interpreters are available, the pool of sign language interpreters tends to be relatively narrow, even in high-income contexts [5]. Forthcoming research suggests that general sign language training does not cover skills to work effectively within the health care context; therefore, issues arise from the limited number of interpreters and their lack of skills [6-8]. Furthermore, health care personnel tend to lack awareness about working with sign language interpreters, alongside limited awareness of deaf communities in general. This results in poor communication, and ultimately, patients do not obtain the information they need to decide on their health or treatment [5].

DHH populations tend to be particularly disadvantaged compared with other persons with a disability. They tend to occupy poorer socioeconomic positions, hold lower health literacy, have insufficient knowledge of health-related vocabulary, and are often unaware of their family medical histories, all of which prevent them from outlining risk factors for their health [9]. DHH individuals have a greater prevalence of obesity, higher levels of hypertension, and higher levels of self-reported depression compared with hearing persons [6,9,10]. There is also a particular concern of underdiagnoses of raised blood pressure and undertreatment of hypertension, diabetes, hyperlipidemia, and cardiovascular disease, among others, due to the lack of effective means of communication between health personnel and deaf patients [6,9-11]. Recent studies claim that by improving communication between deaf persons and nondeaf persons hearing health personnel would have a positive impact on preventive care [12-14].

### Objective

The rapid adoption of VRI technology in health care opens up opportunities to set up more accessible health care. Thus, we need to learn more about how this technology could effectively engage with and respond to the priorities of its users. Emerging literature shows that DHH users tend to prefer in-person to VRI interpretation [15-17]. Furthermore, satisfaction with VRI interpretation tends to be low [15]. We do not have evidence on whether users are comparing interpreters with the same level of skills one via VRI and one in-person, so they are comparing the sentiment of indeed like with like or not. Thus, we need more clarity on the elements of VRI systems that have been examined, such as procedures, available protocols, challenges, and successes. Having detailed data, all elements regarding in-person and VRI interpretation protocols would allow determining the technology that holds some constraints more clearly or the protocol could be improved and made more efficient. It is also necessary to identify the essential elements of VRI as a precondition to encourage rigorous studies and ensure fidelity when implemented. The scoping review approach chosen for this study will allow us to determine the state of available evidence, which is needed before rigorous empirical studies are conducted. Therefore, for the purpose of this study, we used the guidance for conducting systematic scoping reviews by Peter et al [18] to determine the following with respect to the use of VRI in the health care context: does the existing literature provide sufficient evidence on how VRI can enable deaf users to overcome interpretation barriers and improve communication outcomes between them and health care personnel within health care settings?

## Methods

### Overview

In this review, we identified relevant studies in English, Spanish, and French published between 2006 when the first relevant publication in the area was identified and March 2021 in PubMed, Web of Science, Embase, MEDLINE, and Google Scholar. The key search terms used were as follows: Sign language user*s, Deaf, Hard of Hearing, Deafblind and VRI, video remote sign language interpretation, video interpreting service, video conference interpreting and community health, health system, and health personnel. The search also covered all types of health-related activities that are often linked to community health. See search strategies in Multimedia Appendix 1.

### Study Selection

Articles were included for full-text reviews if they were about the use of VRI for DHH users for, or within, health care. Titles and abstracts were screened, and if an article was considered representative of the inclusion criteria, the full text was reviewed. Data extraction was conducted by 2 reviewers, independently, on 20% of the papers. The discrepancies were minimal.

**Figure 1:**
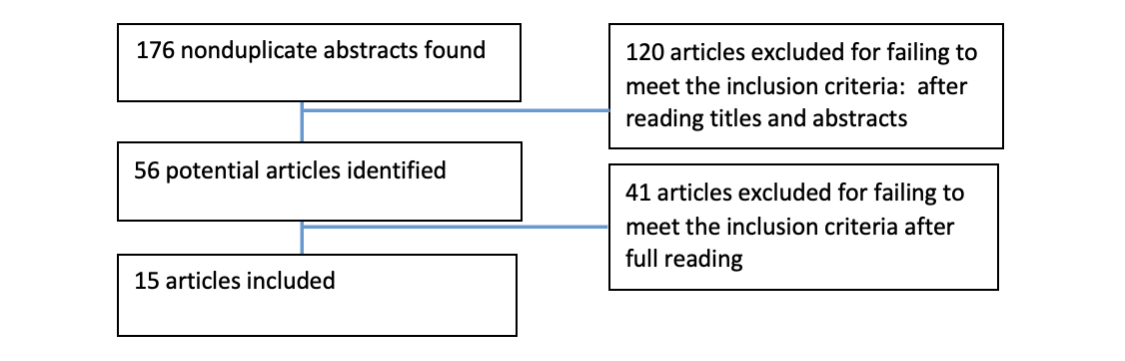
PRISMA (Preferred Reporting Items for Systematic Reviews and Meta-Analyses) flow diagram of study selection.

If the paper was selected for full review, data related to the use of VRI for sign language users within the health care context were extracted. Data extracted from the articles that reported on the analysis, use, or implementation of VRI within the health care context were entered into an Excel (Microsoft Inc) form. Key findings were extracted in a summary format. Information on authorship, publication year, article type, methodology, population, lessons learned, and recommendations regarding the use of VRI were recorded in this form (Figure 1).

### Analysis

We conducted an inductive content analysis of the selected records following the steps outlined by Elo [19]. The extracted findings from each study were subjected to open coding, and similar codes across articles were then identified as concepts coded inductively into the key concepts. Finally, in line with the aims of the study, the concepts were grouped into either the advantages of VRI or the challenges or limitations of VRI.

### Patient and Public Involvement

This study was performed without the involvement of DHH patients. However, it does involve organizations for DHH individuals as well as persons with a disability. The National Deaf Federation of Colombia (FENASCOL) advised MRV on the pertinence of this research. JC, a DHH scientist, has coauthored this paper, contributing to its conceptualization, interpretation of the results, and attainment of clarity and accuracy of the writing.

### Ethics Approval

The research protocol of this study was approved by the ethics committee of the University of Geneva (CUREG_2021-05-50).

## Results

### Overview

From the original 176 articles identified, 120 were eliminated after reading the article title and abstract, and 41 articles were excluded after they were fully read. In total, 15 articles were included in this study: 4 studies were literature reviews, 4 were surveys, 3 were qualitative studies, and 1 was a mixed methods study that combined qualitative and quantitative data, 1 brief communication, 1 quality improvement report, and 1 secondary analysis. Table 1 includes summaries of the articles that met our inclusion criteria.

There is limited research on the use and efficiency of VRI to improve communication between DHH individuals and health personnel within health settings. The current published scientific literature does not allow us to understand either the use of this technology or its impact on quality of care, patient satisfaction, or health outcomes. Nearly half (n=7, 46%) of the articles included empirical evidence on adult DHH VRI users, 1 (6%) on DHH children, 1 (6%) on sign language interpreters, and 1 (6%) on subject matter experts working with older DHH adults. Less than half (n=6, 40%) of the articles explicitly addressed the role of DHH persons as coauthors of the articles and steps followed to fulfill ethical and moral obligations of putting the voice of the DHH population at the center of their research, promoting well-being and the human rights of this population.

A limitation of the available literature is the lack of representation of the DHH population as a whole, given that all the articles are from high-income countries, namely 12 from the United States, 1 from Denmark, 1 from Norway, and 1 from Canada. This is a significant gap, given that 80% of persons with disabling hearing loss live in LMICs [19]. Currently, resource constraints and other social and political barriers in LMICs that could affect the availability, use, and efficiency of sign language interpretation via VRI within health care are not included in the published literature.

The current literature shows the key advantages of pursuing improvements in this technology as well as some recurring challenges and limitations (Textbox 1).

**Table 1:** Summaries of studies included in this review.

Study	Country	Aims	Study population and sample size	Design	Duration of the intervention	Main findings that related to the use of VRI^a^ within health care
Berry and Stewart, 2006 [20]	United States	To outline challenges that D/deaf^b^ people face within health care. It outlines recommendations to ensure a successful medical visit.	D/deaf	Literature review	No information	Suggest capacity building for medical staff regarding communication needs of D/deaf patients. It provides a protocol to identify interpreters, as well as a list of tips for working with an interpreter, such as speaking to patients when using an interpreter.
Steinberg et al, 2006 [21]	United States	To better understand the health care experiences of deaf people who communicate in ASL^c^	Participants were deaf, communication preference for ASL, and willingness to share health care experiences	Qualitative studies (semistructured focus group meetings)	No information	It points out that fear, mistrust, and frustration were prominent in participants’ descriptions of health care encounters, as well as a list of inadequate common practices such as writing notes and using family members as interpreters.
Masland et al, 2010 [1]	United States	This study reviews published literature and unpublished data, documenting the use of telephonic and video interpretation methodologies to improve health care communication.	Published and unpublished literature on the interpretation in health care	Brief communication	No information	This study looks at the cost-effectiveness of VRI for all language translation including sign language. VRI advantages outlined in the study are flexibility, convenience, quality of interpretation, and cost. Some arguments are made that the savings in hiring an ASL interpreter can pay for the installation of video interpretation networks in some hospitals. The results linked the use of VRI to fewer tests, less visits to the hospital, and better treatment adherence. However, evidence represented is in spoken leagues not sign language.
Hommes et al, 2018 [22]	United States	This research aimed to identify ASL interpreters’ perceptions of barriers to effective communication between deaf and HOH^d^ patients and health care providers.	ASL interpreters	A cross-sectional survey	June 15	The results indicated that VRI technology in the absence of an ASL interpreter is considered a better option by many deaf and HOH patients than note-writing or lip-reading; however, the occasional technology malfunctions limit it as a consistently reliable tool.
Dammeyer et al, 2017 [23]	Denmark	This study examined the prevalence of technology use and interpreting services use among people with hearing loss as they relate to demographic characteristics of this population.	269 children (0-15 years of age) and 839 adults (16-65 years of age)	National surveys of children and adults with hearing loss	2014	This study found that sign language users, both children and adults, prefer VRI over other communication technology. Adults with a bachelor’s degree or higher reported more frequent use of mobile video interpretation and texting devices. This study underlines the need for a user-centered approach and user involvements to address environmental and personal factors affecting assistive technology use. It recommends that deaf people may benefit from accessing well-trained personnel who understand the individual’s needs and facilitate technology-person match.
Myers et al, 2021 [16]	United States	To examine the extent to which communication aids and services used by ASL users and their health care providers aligns with preferences, satisfaction, and unmet needs and to elicit from stakeholders’ strategies to address disparities	ASL users in North Carolina	Web-based survey (cross-sectional study)	May 2018 until March 2019	The study found that accessible communication was associated with 81% lower odds of dissatisfaction with communication. Better communication was linked to better relationships with the health providers. The study claims that improving communication would have a positive impact on preventive care. The study identifies several issues with the use of VRI. One of the most common barriers to accessible communication via VRI were technical problems, as well as quality of sign language interpreting services. Communication via VRI was considered not user-friendly, creating frustrations for both deaf individuals and their professional health care providers. Health providers attempted to adapt to VRI issues by lipreading or speech or writing notes back-and-forth, both methods were inadequate and did not lead to improved communication. The study made specific technical recommendations on when and how to use VRI in clinical settings.
Kushalnagar et al, 2019 [15]	United States	This study aimed to investigate the national trends of deaf patients’ satisfaction with the quality of VRI in health settings and recommend actions to improve VR quality and deaf patients’ satisfaction with VRI in health care settings.	Persons that use ASL as a primary language, age of 18 years or above, and presence of bilateral hearing loss	Secondary Analysis of National health trends Survey in ASL	Between 2016 and 2018	The study shows that almost half of the people reached by the survey did not have access to VRI over the last 12 months. It also shows that those who have access were largely dissatisfied with the quality of the service. About 41% (n=228) of the deaf patient sample rated the quality of VRI as satisfactory. The rest (n=327, 59%) rated their VRI experience as unsatisfactory. VRI tends to be cost-effective and its flexibility is of great advantage to service providers, users, and interpreters. The study claims that if D/deaf ASL health care users are provided with a fully functioning VRI system with qualified interpreters, this system can potentially reduce the number of emergency visits and unnecessary diagnostic tests, all of which are associated with cost burden.
Yabe, 2020 [17]	United States	This study identifies health care providers’ and DHH^e^ patients’ interpreting preferences for VRI and in-person interpretation during critical care and noncritical care	1. Health care providers who had used VRI in clinical settings in the past 10 years were 18 years or older and spoke English. 2. DHH patients who had used VRI in clinical settings in the past 10 years were 18 years or older and used ASL	Mixed methods design incorporating both an online survey and qualitative interviews	No information	This study provides the views of both health workers and sign language users—the findings pointed out that VRI is the preferred way of communication of patients and health providers for noncritical care. VRI offers preparedness unattainable with in-person interpretation. Furthermore, in-person interpretation is limited in its availability and represents at times economic loss. It outlines technical limitations regarding VRI and recommendation for its use. It points out that patient’s acceptance of VRI was linked to time constraints and type of care. Thus, acceptance was limited as it was described as waste of money as it did not prove effective for communication. For providers, its convenience and flexibility were very important.
Kushalnagar et al, 2017 [24]	United States	The objectives of this study are (1) to culturally adapt and linguistically translate the HINTS^f^ items to ASL (HINTS-ASL) and (2) to gather information about deaf people’s health information–seeking behaviors across technology-mediated platforms.	Deaf adults (ages 18-90 years and above) who use ASL	Qualitative studies (cognitive interviews)	N/A^g^	This article outlines the protocol of cultural adaptation national survey items exploring VRI. Linguistic adaptation of items related to time, explanation of illness and use of diagrams, captions and videos is very useful for validation studies using sign language.
Singleton et al, 2019 [25]	United States	This study explored technology use among older deaf adults with regard to attitudes, adoption style, and frequency of use for a wide range of technologies, including ATs^h^ for persons with hearing loss and general everyday technologies.	Participants had to be 50 years of age or older and self-identify as DHH	Online or paper copy questionnaire	—^i^	Older adults are moving away from TTYj and TDDk to embrace VPsl and VRSm; 51% of respondents use VRI. They noticed that consumer service and support such as free delivery and personnel to set technology up had a very positive impact on the consumer experience. Participants reported difficulty keeping up with software updates and other technology maintenance activities that require a higher level of computer literacy. Thus, many older adults in the deaf community appear to be comfortable with daily technologies and ATs and especially video-based internet technologies that support communication accessibility such as VP and VRS.
Kasales et al, 2020 [26]	United States	The goal of this review is to help members of the breast center team better understand (1) the mandates of the ADA^n^ and the challenges faced by patients with select communication disabilities.	Descriptive review	Literature review (descriptive review)	N/A	This article reviews some relevant literature and points out recommendations to use VRI. However, it does not include any empirical evidence. They recommend using VRI when an in-person interpreter is not available and only in agreement with the patient. It lays out the recommendation of the National Association of the Deaf Seniors of America for the use of VRI for ASL communication.
Meulder and Haualand, 2019 [27]	Norway	To critically assess the impact and role of SLIS^o^ in those countries where SLIS have been institutionalized	VRI deaf users	Literature review (conceptual analysis)	N/A	This article presents an analysis of the role that sign language interpretation has in social services including health care. The paper makes a strong argument for the importance of language-concordant services. It does refer broadly to sign language interpretation including VRI. It highlights that access and communication in the health care setting are mainly conceptualized and arranged with a hearing person’s perspective. Little has been done to allow health settings or personnel to be bilingual and therefore more accommodating to the sign language users, cultural gaps, discriminatory set up, and other issues might not be apparent to the interpreter and shall be considered.
Preusse et al, 2016 [28]	United States	The goal of this study was to identify the range of challenges in everyday activities that might be experienced by older adults aging with preexisting impairments in vision, hearing, or mobility.	Interviews with subject matter experts working with older deaf adults	Qualitative study (interviews)	—	Findings of the study revealed challenges faced by deaf persons as they age. These challenges include access to social services, adequate housing, and technology. The findings state that access to interpreters is an issue in most health settings. Experts interviewed pointed out that this shortage of qualified sign language interpreters can be overcome by using VRI. Thus, they also pointed out that VRI may be inappropriate when people are dealing with high levels of stress such as a medical emergency. In these cases, in-person interpretation may be more appropriate, if available. The findings show that device maintenance and software updates are difficult for this population. The study recommends one-to-one training for uptake of new technologies, as well as mixed available technologies such as haptic devices as medication reminders.
McKee et al, 2015 [29]	United States	The aim of this paper is to summarize evidence and good practices on how to enable better communication between DHH and health personnel, particularly physicians.	—	Literature review	N/A	This paper offers an overview of good practices and questions regarding health service provision for DHH patients. It lays out that DHH patients are more likely to experience poverty and less likely to access ICTp including smartphones. VRI is mentioned as a tool to overcome communication barriers and improve satisfaction, quality of care, and health outcomes. However, it also mentioned that evidence on the impact of interpretation and VRI is lacking. These recommendations assume that interpretation availability either via VRI or in person is an efficient way forward.
Kwok et al, 2021 [30]	Canada	This report documents the experience in using web-based technology in an emergency department to meet communication needs of our patients who have LEP^q^ including deaf sign language users during the COVID-19 pandemic.	—	Quality improvement report	March 30 and May 31, 2020	This study focuses on the use of VRI more generally for patients of linguistic minorities including sign language. It reports on the cost-efficiency of the intervention, laying out prices of VRI inclusive of sign language and claiming that such a cost is not problematic to absorb by the hospital. VRI technical issues were easily overcome and personnel became acquainted to its use relatively easily. Furthermore, the study claims that the use of VRI also complies with security protocols in place during the COVID-19 pandemic and allows the protection of interpreters and others from exposure. The authors of the paper judged that this intervention was successful for both hearing patients and DHH patients. Thus, there is no evidence that it was the case.

^a^VRI: video remote interpretation.

^b^D/deaf: “Deaf” refers to the linguistic minority while “deaf” refers to persons with hearing impairment.

^c^ASL: American Sign Language.

^d^HOH: hard of hearing.

^e^DHH: deaf or hard of hearing.

^f^HINTS: Health Information National Trends Survey.

^g^N/A: not applicable.

^h^AT: assistive technologies.

^i^Data not available.

^j^TTY: (teletypewriter) is a communication device used by people who are deaf, hard-of-hearing, or have severe speech impairment.

^k^TDD: test-driven development.

^l^VPs: videophones.

^m^VRS: video relay service.

^n^ADA: Americans with Disabilities Act.

^o^SLIS: Nottinghamshire Sign Language Interpreting Service.

^p^ICT: information and communications technology.

^q^LEP: limited English proficiency.

**Summary of advantages and disadvantages.**

**Advantages**

- Convince
- Preparedness unattainable with in-person interpretation
- Access to qualified interpreters
- Possibility to work remotely for interpreters
- Safety, limiting social contact in health care environment
- Cost
- Flexibility

**Disadvantages**

- Technology malfunctions
- Inaccessible to deaf patients in certain physical positions and those with vision impairment
- Requires higher level of computer literacy
- Not user-friendly
- For some it might limit patient-provider relationship
- Relays on the availability of reliable internet access and adequate devices

### Advantages of Using VRI Interpretation

Early literature [1,20,21] described sign language interpretation using VRI in health care settings as equally efficient as in-person interpretation. Advantages attributed to the technology, such as flexibility and affordability, encourage the idea that this technology could help overcome the shortage of qualified sign language interpretation in health care settings. It also pointed out that the use of VRI could help to override the use of inadequate techniques such as lipreading and note-reading, which are often used in health consultations with DHH patients. DHH sign language users prefer to use VRI over these techniques primarily because it allows them to communicate in their preferred language, sign language [22,26]. Lipreading and note-reading often assume that sign language users are proficient in reading and writing in a spoken language, which is often not the case. The literacy rates of DHH communities are at a sixth grade reading level or lower [29,31,32].

Articles exploring technology preferences highlight that sign language users (both children and adults) prefer VRI to other communication technology over texting devices (sign language, text, and speech interpretation linked by a call center or voice recognition technology) [16,23]. As the proliferation of VRI technology increases, consumer choices increase. With this technology, deaf patients have the possibility to choose communication tools and assistance that they deem more appropriate for their medical consultation [15-17,27]. For some noncritical medical services, VRI is preferred over in-person interpretation [16,17,24].

Sign language interpreters saw a significant advantage to this technology as it allowed them to eliminate time for transportation, given that most of their time assisting in a medical consultation is consumed by traveling to the location [22]. Saving in traveling time often translates to saving in the total cost of the interpretation. This is a key advantage often mentioned in the literature and an underlying motivation to continue expanding the use of VRI in health care settings [1,15,17,20-22,25,28,30]. VRI has also proven advantageous during the COVID-19 pandemic, allowing qualified interpreters to be available at emergency services while protecting both parties from risking potential exposure at the emergency room and complying with access restrictions [30].

The current literature suggests that the use of VRI to use qualified sign language interpreters, despite where they are located, has the potential to help overcome the scarcity of sign language interpretation and enable better communication between deaf patients and health care personnel. The advantages offered by VRI are likely to be enhanced as technology devices such as tablets, laptop computers, and smartphones become more affordable and reliable internet bandwidth becomes more available [15,17,25].

### Challenges and Limitations of the Technology

As evidence grows, we are learning more about VRI technology because of its shortcomings, particularly with regard to the specificities of health care settings. A national survey conducted in the United States showed that only almost half of the representative sample did not have access to VRI during health care appointments over the last 12 months [15]. It is not clear whether the technology was needed but not available, suggesting that even in a high-income context, the availability of this technology remains limited or if participants chose not to use VRI because they had access to in-person interpretation or preferred to use other communication techniques.

Several articles in the hospital context in the United States showed that VRI was not user-friendly and led to frustration for both DHH individuals and their professional health care providers. The most common barriers noted were technical problems and poor quality of sign language interpreting services [16]. Although VRI is preferred for noncritical care, it is considered inappropriate for critical care or stressful situations [16,25]. A second article reiterated the issues found in the first study and laid out other technical issues, such as limited placement and positioning of devices, negatively impacting the experience of using this technology [17]. They also found that VRI was seen as inconducive to enriching patient-health provider relationships and that providing VRI without previously notifying, seeking, and obtaining the agreement of the patient first was bothersome [17].

A national survey from the United States also looked at preferences of the DHH population between VRI or in-person interpretation within health care settings and found that 59% of their respondents rated their VRI experience as unsatisfactory and preferred in-person interpretation. Sign language interpreters have also reported concerns regarding technology. According to interpreters’ views, in-person interpretation is more efficient at identifying when users do not understand a diagnosis, medical instructions, or other information compared with VRI. Interpreters also pointed out that the extra time before and after the appointment is useful for reviewing information available in the lobby and preparing for consultation, which enables them to provide better interpretation services [22]. The VRI does not allow interpreters to prepare or debrief DHH patients before and after consultations [31]. In turn, VRI could be more prone to incomplete communication between DHH and hearing health personnel. Capacity building among health personnel was noted as a significant communication barrier for DHH patients but also as a hindrance to technology development [20].

The efficiency of this technology is partially determined by the appropriateness of the video device used. The recommended screen of a minimum of 49.5 cm (19.5 in) is often not available [26]. Keeping up with software updates among other technologies, maintenance was considered burdensome among older DHH adults [25]. Other reported limitations of the technology included constraints due to the physical position of the patient. VRI is not accessible for patients undergoing clinical examination that requires them to be face down; VRI is also not accessible for DHH persons who are blind or have low vision [16]. The use of electronic means of communication for health information also raises security and privacy concerns. We found no information on whether the video feeds were encrypted.

The literature also shows methodological shortcomings of using health research instruments, such as surveys that explore VRI on DHH individuals, which have been developed and tested only with hearing participants. Given the cultural and linguistic differences between DHH and hearing populations, some concepts, questions, and wording may be inappropriate or incomprehensive for DHH individuals [24].

Adding to the technical and methodological issues, a more troubling challenge was assuming that an efficient VRI technology would be sufficient to overcome barriers to health care for DHH individuals (or communities). Research has shown that the use of VRI services alone is not fully accessible to DHH communities. Little research has been conducted to promote bilingualism or language-concordant practices across health settings or personnel and accessibility in broader health-related communication practices [27]. Furthermore, there is a risk that the VRI could be conceptualized and put in place from a hearing person’s perspective. This limited, 1-sided view ignores issues related to cultural differences and discrepancies, discriminatory practices, intrinsic bias, and intersectionality issues related to hearing status, ethnicity, race, or multiple disabilities.

## Discussion

### Principal Findings

This scoping review provides an overview of the current evidence on the efficiency of the use of VRI with deaf users within health care settings. It shows that this area is under research, and the evidence is scant. It is particularly concerning that all articles found were from high-income countries, given that most DHH people live in LMICs. There is a dearth of evidence on the use of VRI and its efficiency and potential across LMICs. This reflects the long-lasting absence of voices of persons with disabilities from non-Western nations on both disability scholarship and technology innovation [33-35]. The lack of knowledge regarding the needs and realities of DHH individuals in LMICs extends beyond VRI technology. Technological progress has often overlooked the experience of disability and the everyday needs and constraints of DHH persons from the Global South. Nearly all research on assistive technology and ICT accessibility for DHH individuals and for persons with disabilities, whether from the legal, technical, or development fields, has focused on high-income countries and very little to no attention has been paid to LMICs [36]. Technological progress has often overlooked the experience of disability and the everyday needs and constraints of persons with disabilities from the Global South, among other reasons, because it is perceived as nonprofitable [34]. Failing to address this gap will cause persons with disabilities in LMICs to continue to be left behind in relation to universal health coverage.

At present, 164 countries are signatories to the Convention on the Rights of Persons with Disabilities (CRPD). CRPD Article 25 on health and Article 9 on accessibility provided the legal basis for ensuring the right to the highest attainable standard. Thus, the implementation of the CRPD remains limited, particularly in LMICs. The dominant presence of the literature from the United States may be linked to the Americans with Disabilities Act of 1990 [37], which lays the legal grounds for accessibility and nondiscrimination, as well as for the adoption of reasonable accommodation. However, similar legal frameworks have been adopted in other high-income countries with sufficient infrastructure to provide VRI services, such as the Disability Discrimination Act 2005 [38] in the United Kingdom, and we did not observe the same level of engagement on behalf of either public health or disability scholars. Nevertheless, the implementation of such CRPD rights to health and accessibility in health care settings will require robust evidence regarding the priorities, needs, and constraints of persons with disabilities in LMICs.

A major strength of this review is the use of a comprehensive search in 3 languages in a rapidly expanding technology and a focus on highlighting available evidence and gaps. A key issue highlighted by the available literature is that the availability of VRI technology has the *potential* to address communication barriers within the health care setting, in addition to other available services and tools aside from, inter alia, in-person interpretation, telephone typewriters, and telecommunications relay services. The views, needs, and rights of the DHH community should be at the core of the development of these technologies. However, the VRI is not a quick fix to overcome accessibility issues [15,27,39]. It is important that its expansion and convenience do not undermine the possibility for DHH communities to choose whichever means of communication they prefer or which is more appropriate for the type of care they seek.

This review also pointed out a knowledge gap regarding the quality of interpretation and training in sign language interpretation for health care. It is not clear if poor-quality interpretation is a recurring issue when using in-person interpretation or if it is only an issue when using VRI [15-17]. There are no data on whether in-person interpretation, as requested in advance, the assigned interpreter is likely to use time before the consultation to undergo a prescreening for interpretation competencies, allowing better preparation for their job. Perhaps interpreters are better matched at the time of assigning the task; thus, we do not know whether this could improve the quality of interpretation. Nor do we know if such prescreening for qualification takes place for VRI interpreters or if such practice would lead to better outcomes and positive experiences across DHH users. There is a gap in the evidence on this issue, although most articles mentioned the pertinence of training for sign language interpreters on health interpretation for better communication outcomes.

The challenges documented in the literature highlight recurring technical issues regarding internet reliability, availability, and adequacy of devices in hospital settings. Although the internet is growing globally [40], it is clear that internet reliability has imposed utmost complex infrastructural challenges that could hamper VRI development in LMICs. The literature is not clear on whether, when VRI is used, users use their own devices or if they have to personally purchase internet data (and devices). This raises questions and concerns, as persons with disabilities are more likely to experience poverty in both high- and low-income countries. The financial challenges of DHH communities will have an impact on access to devices and the internet, and in turn, these challenges will impose further barriers to communication and health care. This is perhaps more acute in the Global South.

For future research, there is a need to raise awareness and build capabilities across health systems to improve accessibility for DHH individuals. The literature suggests that having more bilingual health workers, language-concordant services, better technologies, and raising awareness will contribute to better communication between DHH communities and health personnel [41-47]. New developments include technologies such as intelligent personal assistants such as Alexa, which can be used with sign language to improve communication [48]. Thus, we need to learn more about how to make health systems more accessible to DHH individuals. Accessible communication in health settings has been linked to fewer hospital visits, better treatment adherence, more cancer screening, and better oral health [10,12,14,41,42,49,50].

### Comparison With Previous Literature

There have been no similar publications in this area. This study provides a well-needed analysis regarding knowledge gaps and the need for future research on the efficiency of VRI technology for sign language users in the health care context.

### Limitations

Our study has a few limitations. We looked at articles examining VRI in health care settings, including hospitals, preventive care, and community health. Few rigorous articles have studied VRI for sign language users in the health care context. The protocols used and examined regarding the use of VRI for sign language are not generalizable at a national level or international level. We attempted to map and assess the available scientific literature.

### Conclusions

The available literature shows that VRI may enable deaf users to overcome interpretation barriers and can potentially improve communication outcomes between them and health personnel within health care services. Communication between DHH health care users and personnel shall improve if sign language users are provided with a VRI system supported by devices with large screens and a reliable internet connection, as well as qualified interpreters trained on medical interpretation. Perhaps issues regarding lack of preparation for interpreters could be overcome by providing VRI interpreters with a brief summary of the purpose of the visit, as well as the background of the consultation before the discussion. Such preparation may allow interpreters and users to develop a rapport during health visits, and research is needed in this area.

Furthermore, our understanding of the use of VRI technology is pertinent and relevant. All articles mentioned that sign language interpretation is a scarce resource within health care systems, even in high-income countries. Thus, learning more about the possibilities and limitations of VRI is even more urgent in LMICs, because the dearth of data and in-person interpretation are largely unavailable and perhaps unfeasible in the near future in resource-constrained contexts.

## Appendix

Multimedia Appendix 1 Search strategy.

## Abbreviations

CRPD: Convention on the Rights of Persons with Disabilities
DHH: deaf or hard of hearing
FENASCOL: National Deaf Federation of Colombia
LMIC: low- and middle-income country
VRI: video remote interpretation
