# Supplementary material for: Video Relay Interpretation and Overcoming Barriers in Health Care for Deaf Users: Scoping Review
Source: J Med Internet Res. 2022 Jun 9;24(6):e32439. doi: 10.2196/32439 (PMC9227653; doi:10.2196/32439)
Supplement: Multimedia Appendix 1 [file jmir_v24i6e32439_app1.docx]

Multimedia Appendices A

Search Strategy

**PubMed**

([([(“Deafness” [Mesh]) OR “Sign Language” [Mesh]] OR [([(deafness[Title/Abstract]) OR (language sign user[Title/Abstract])] OR [deaf patient[Title/Abstract]]) OR (hearing loss[Title/Abstract])]) OR (deaf)AND ([([(video relay service*[Title/Abstract])] OR [video remote interpret*[Title/Abstract]]) OR (Videotelecommunication remote interpret*[Title/Abstract])] OR [assistive technology(Title/Abstract)])])

**Embase**

('deaf patient':ab,ti OR deafness:ab,ti OR 'hearing impairment':ab,ti OR 'hard of hearing':ab,ti OR 'hearing loss':ab,ti OR 'persons with hearing impairment*':ab,ti OR 'sign language*':ab,ti OR 'hearing disorder*':ab,ti OR 'hard of hearing'/exp OR 'sign language'/exp) AND ('communication aid'/exp OR 'assistive technology'/exp OR 'interpreter service'/exp OR 'online system'/exp OR 'video relay service*':ab,ti OR 'video remote sign*':ab,ti OR 'videotelecommunication*':ab,ti OR 'video remote interpreting*':ab,ti OR 'assistive technology':ab,ti OR 'video remote sign':ab,ti OR 'video interpret*':ab,ti OR 'communication aid*':ab,ti OR 'interpreter service*':ab,ti OR 'online system':ab,ti)
